# Deep learning-based radiomics does not improve residual cancer burden prediction post-chemotherapy in LIMA breast MRI trial

**DOI:** 10.1007/s00330-025-11801-z

**Published:** 2025-08-06

**Authors:** Markus H. A. Janse, Liselore M. Janssen, Elian J. M. Wolters-van der Ben, Maaike R. Moman, Max A. Viergever, Paul J. van Diest, Kenneth G. A. Gilhuijs

**Affiliations:** 1 Image Sciences Institute, University Medical Center Utrecht, Utrecht University, Utrecht, The Netherlands; 2 Department of Radiology, St. Antonius Hospital, Nieuwegein, The Netherlands; 3 Department of Radiology, Alexander Monro Hospital, Bilthoven, The Netherlands; 4 Department of Pathology, University Medical Center Utrecht, Utrecht University, Utrecht, The Netherlands

**Keywords:** Breast MRI, Neoadjuvant chemotherapy, Response assessment, Residual cancer burden, Deep radiomics

## Abstract

**Objectives:**

This study aimed to evaluate the potential additional value of deep radiomics for assessing residual cancer burden (RCB) in locally advanced breast cancer, after neoadjuvant chemotherapy (NAC) but before surgery, compared to standard predictors: tumor volume and subtype.

**Materials and methods:**

This retrospective study used a 105-patient single-institution training set and a 41-patient external test set from three institutions in the LIMA trial. DCE-MRI was performed before and after NAC, and RCB was determined post-surgery. Three networks (nnU-Net, Attention U-net and vector-quantized encoder-decoder) were trained for tumor segmentation. For each network, deep features were extracted from the bottleneck layer and used to train random forest regression models to predict RCB score. Models were compared to (1) a model trained on tumor volume and (2) a model combining tumor volume and subtype. The potential complementary performance of combining deep radiomics with a clinical-radiological model was assessed. From the predicted RCB score, three metrics were calculated: area under the curve (AUC) for categories RCB-0/RCB-I versus RCB-II/III, pathological complete response (pCR) versus non-pCR, and Spearman’s correlation.

**Results:**

Deep radiomics models had an AUC between 0.68–0.74 for pCR and 0.68–0.79 for RCB, while the volume-only model had an AUC of 0.74 and 0.70 for pCR and RCB, respectively. Spearman’s correlation varied from 0.45–0.51 (deep radiomics) to 0.53 (combined model). No statistical difference between models was observed.

**Conclusions:**

Segmentation network-derived deep radiomics contain similar information to tumor volume and subtype for inferring pCR and RCB after NAC, but do not complement standard clinical predictors in the LIMA trial.

**Key Points:**

***Question*** *It is unknown if and which deep radiomics approach is most suitable to extract relevant features to assess neoadjuvant chemotherapy response on breast MRI*.

***Findings*** *Radiomic features extracted from deep-learning networks yield similar results in predicting neoadjuvant chemotherapy response as tumor volume and subtype in the LIMA study. However, they do not provide complementary information*.

***Clinical relevance*** *For predicting response to neoadjuvant chemotherapy in breast cancer patients, tumor volume on MRI and subtype remain important predictors of treatment outcome; deep radiomics might be an alternative when determining tumor volume and/or subtype is not feasible*.

**Graphical Abstract:**

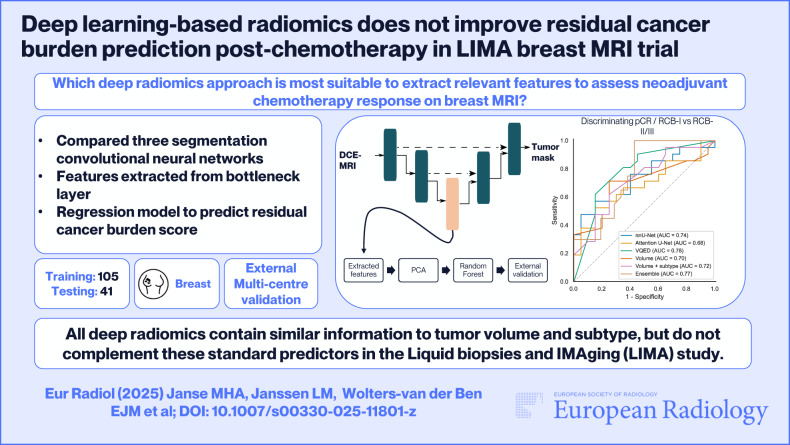

## Introduction

Neoadjuvant chemotherapy (NAC), where chemotherapy is administered prior to surgery, is becoming an increasingly common treatment strategy for patients with locally advanced breast cancer (LABC). The complete absence of microscopic tumor cells in the resection specimen after NAC is known to be correlated with superior overall and recurrence-free survival [1]. In addition to pathological complete response (pCR), residual cancer burden (RCB) has also been shown to be associated with event-free survival [2, 3].

Recent studies have focused on minimizing or omitting surgery if pCR is anticipated [4, 5]. Thus, it is of considerable interest to explore predictors of tumor response to NAC prior to surgery. However, reliable predictors of pCR have not yet been identified. Dynamic contrast-enhanced magnetic resonance imaging (DCE-MRI) is considered the most sensitive method to visualize breast lesions in three dimensions [6], but neither DCE-MRI nor other imaging modalities such as ultrasound or mammography offer sufficient specificity to reliably identify pCR [7–10]. Even targeted biopsies at the tumor site were not found to increase accuracy sufficiently to safely omit surgery due to sampling error [5].

It is currently unknown whether the tumor-occupying region on MRI harbors other factors invisible to the naked eye that could provide additional value to conventional biomarkers of tumor response, such as tumor volume. Several studies have attempted to use machine learning to predict or assess response to NAC on DCE-MRI. Methods generally fall into two categories: radiomics, where a set of hand-crafted features are used, and deep learning, which attempts to learn the best representation from the imaging data. Most studies have limited clinical applicability due to validation being restricted to older and/or single-institution data, or the inability to demonstrate complementary value over conventional clinical parameters [11–13].

The field of deep learning-based radiomics, or deep radiomics, represents a more recent paradigm that has gained considerable interest [14]. Deep radiomics combines proven machine learning techniques with automated feature extraction using deep learning to analyze imaging data. While traditional radiomics requires time-consuming manual feature engineering, deep radiomics extracts a representative feature set fully automatically. Currently, there is no consensus on which deep radiomics approach is most suitable to extract relevant features from imaging, nor is their performance known for assessing NAC response on breast DCE-MRI.

This study aimed to evaluate the predictive value of deep radiomics for assessing RCB in locally advanced breast cancer after NAC, but before surgery, compared to standard clinical predictors such as tumor volume and subtype.

## Materials and methods

### Subjects

Two cohorts were used in this retrospective study: a single-institution cohort for training and a multi-institution cohort for testing and validation. The training set was acquired retrospectively. The requirement for informed consent and ethical review was waived by the institutional review board (IRB) (Medical Research Ethics Committee Utrecht [METC Utrecht], no. 19-245). The test set was acquired in the context of a prospective clinical trial, which was approved by the IRB (METC Utrecht, no. 19-396), and informed consent was obtained from all participants. Both datasets were previously described in detail in [15], and are briefly summarized below.

The training set consisted of patients with stage 1–3 invasive breast cancer who were treated with NAC followed by surgery at the University Medical Center Utrecht (UMC Utrecht) between January 2011 and December 2019. Patients with oligometastatic disease were included if they received treatment with curative intent. Exclusion criteria were: external, incomplete or corrupt diagnostic imaging, major deviations from treatment guidelines, recurrence of a previous malignancy, simultaneous systemic treatment for a non-breast malignancy, and the presence of foreign objects in the breast region during imaging (excluding radiological markers).

The test set consisted of patients from the Liquid biopsies and IMAging (LIMA) study [16]. Patients with histologically proven invasive breast cancer scheduled for NAC were included from four institutions between December 2019 and October 2021. Exclusion criteria were: luminal A subtype, inflammatory breast cancer, any distant metastases at the time of diagnosis, prior history of ipsilateral breast cancer, and contraindications to DCE-MRI. To avoid overlap with hospitals in the training set, patients from UMC Utrecht were excluded from the test set.

### MR imaging

DCE-MRI was performed on all patients. Two examinations were used in this study: an examination at baseline prior to any NAC (“pre-NAC”), and one prior to surgery (“post-NAC”). The second DCE-MRI examination was performed after either all cycles of NAC were received prior to surgery, or after five out of six cycles of NAC, depending on the patient’s NAC schedule. All examinations in the test set were centrally revised by a dedicated breast MR radiologist (E.J.M.W.v.d.B., 20 years of experience) following the Breast Imaging Reporting and Data System (BI-RADS) descriptors, as previously described [17, 18].

The DCE-MRI protocol consisted of five post-contrast series in the training cohort and a minimum of three post-contrast acquisitions in the test set, taken at a 60–90 s interval. Only the T1-weighted dynamic contrast series were analyzed in this study. Acquisition parameters are detailed in Table 1. In the training cohort, imaging was performed using either 1.5-T or 3-T MRI scanners from a single vendor (Philips), while in the test set, imaging was exclusively performed on 3-T scanners from multiple vendors. Fat suppression was applied to all scans.

**Table 1:** MRI characteristics of the training and test set

MRI parameter	Training set	Test set
Field strength	1.5 T, 3 T	3 T
MRI scanner model	Philips Achieva, Ingenia	Philips Ingenia; Siemens MAGNETOM Avanto, Spectra, Skyra or Vida
Contrast agent	Gadovist (Bayer)	Dotarem (Guerbet); Gadovist (Bayer); Prohance (Bracco)
Voxel volume (mm³)	0.75 × 0.75 × 0.90–0.97 × 0.97 × 1.30	0.5 × 0.5 × 0.9–1.0 × 1.0 × 1.25
Interval between contrast series (s)	60–90	55–89
Fat suppression	Yes	Yes
Flip angle (°)	8–10	8–12
Echo time (TE) (ms)	1.2–3.4	1.5–2.5
Repetition time (TR) (ms)	3.3–7.1	3.7–5.5
Acquisition plane	Axial	Axial

### Histopathologic resection specimen evaluation

All histopathological resection specimens were centrally revised by a dedicated breast pathologist (P.J.v.D., 30 years of experience) to assess RCB [19]. Cases where resection specimens were not available were assigned an RCB score of 0.0 in the case of pCR. Cases with reported residual invasive disease but no available resection specimens were excluded. RCB was divided into four categories as described by Symmans et al For further analysis, categories RCB-0 (= pCR) and RCB-I (minimal residual disease) were classified as responders, while categories RCB-II and RCB-III (moderate to extensive residual disease) were classified as non-responders. Based on the receptor profile of the pre-NAC biopsy, tumors were subtyped as triple negative (TN) for the estrogen (ER), progesterone and HER2 receptors, ER+/HER2- or HER2+ as before [15]. The percentage of tumor-infiltrating lymphocytes (TILs) was assessed by two experienced breast pathologists as previously described [20].

### Image pre-processing

Images were automatically cropped in the anterior-posterior direction between 1 cm anterior of the nipple and 5 cm posterior of the intermammary cleft using the method described in Verburg et al [21]. Subsequently, all post-contrast DCE-MRI were registered to the pre-contrast acquisition using *elastix* [22]. After registration, images were resampled to the median voxel spacing of the training set. No bias correction was applied.

### Ground truth tumor segmentation

Ground truth tumor segmentations were obtained using semi-automatic region growing, following a previously described procedure [15, 23]. In short, one or multiple seed points were placed in or near the tumor center, followed by constrained volume growing. Any erroneously segmented blood vessels were manually removed. Seed points were placed and checked based on clinical radiology reports by a biomedical engineer (M.H.A.J., 5 years of experience in breast MRI), under the supervision of a breast MR radiologist (E.J.M.W.v.d.B., 20 years of experience).

### CNN training

Three convolutional neural networks (CNNs) trained for tumor segmentation were used to calculate deep features: nnU-Net [24], Attention U-Net [25], and a vector-quantized encoder-decoder (VQED) [26]. We chose these networks for the following reasons: (1) nnU-Net is one of the most popular medical segmentation frameworks, with a wide variety of available pre-trained networks, (2) Attention U-Net is a similar U-Net-derived architecture (with added attention gating) but has a completely independent implementation, thus mitigating any potential issues related to the implementation of nnU-Net, and (3) VQED is a non-U-Net-derived network that has previously shown promise in deep feature extraction.

The input to the networks consisted of the T1-weighted pre-contrast series and multiple post-contrast series, with the target output being a segmentation mask of the tumor. The Dice score was used to assess segmentation performance on the test set, limited to the symptomatic breast region [15]. In case less than five post-contrast series were available, the last series was carried forward to obtain five series in total.

The first CNN was a previously trained, publicly available CNN based on three-dimensional nnU-Net [15, 24], taking as input one pre-contrast and five post-contrast series. This previous study used the same training dataset as the current study. The second CNN was a two-dimensional attention-gated U-Net [25], taking as input all slices of the pre-contrast, first post-contrast, and fifth post-contrast series. Details regarding the training procedure of Attention U-Net are described in the Supplementary Information. The third and final CNN was a vector-quantized encoder-decoder (VQED) network based on the work of Kobayashi et al [26]. The input to this two-dimensional network were also axial slices of pre-contrast and five post-contrast series. All networks were trained using their own default data augmentation parameters.

### Deep features calculation

**Fig. 1:**
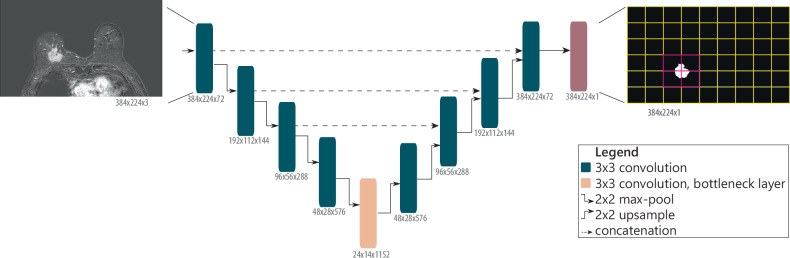
Illustration of deep radiomics extraction from U-Net-based networks, shown here for the Attention U-Net. Activations at the bottleneck layer (bottom rectangle, in light orange) are extracted based on the tumor segmentation (right). The yellow grid illustrates the coarse resolution of voxels in the bottleneck layer. Deep features are extracted from the magenta voxels containing tumor segmentation mask. Below each convolution layer, the output dimensions are stated. The attention blocks are not shown in this diagram

Segmentation networks typically down-sample the input image into a more compact representation, before up-sampling back to the original image size. The most down-sampled layer, called the bottleneck layer, harbors the most compact representation of the image necessary for segmentation. We define the individual neural activations in this layer as “deep features,” which are the activations of a given channel at the bottleneck layer. These deep features were then combined into one feature activation vector for subsequent modeling. Deep features were extracted from the bottleneck layers based on corresponding voxels in the segmentation output. This is illustrated in Fig. 1. Technical details on the feature extraction are described in the Supplementary Information.

Deep features were extracted separately for each examination (pre-NAC and post-NAC series), and a set of delta-NAC features was calculated by subtracting the post-NAC features from the corresponding pre-NAC features.

### RCB prediction

Six random forest regression models were trained to predict the RCB score: three deep radiomics models (one for each neural network), two models using only routine clinically available information (i.e., the ‘clinical-radiological models’), and an ensemble (combined model) of the neural network models with one clinical-radiological model (the most extensive model). We detail the training procedure, including dimensionality reduction using principal component analysis (PCA), in the Supplementary Information. Of note is that optimal hyperparameters for the random forest regression model were determined using five-fold cross-validation on the training set.

Two clinical-radiological models were trained to serve as baseline reference. The first model used two input features related to tumor volume: post-NAC tumor volume (in mm³) and the difference in volume compared to baseline (in mm³), both established from tumor segmentation. The second model also took tumor subtype into account (either TN, ER+/HER2- or HER2+) [15]. PCA was not applied to these features.

The ensemble model was constructed and tested for possible complementary value: a linear regression model was fit to predict RCB based on the predicted RCB output of the three deep radiomics models and the clinical-radiological model with both volume and subtype. The linear regression was constrained to have positive coefficients with zero intercept. The coefficients of the linear regression model were then extracted to determine the individual impact of each model on the final output of the ensemble.

### Statistical evaluation

The primary endpoint of this study was the RCB score. The correlation between predicted and actual RCB scores was assessed using Spearman’s $\rho$. Based on the predicted RCB score, the area under the receiver operating characteristics curve (AUC) was calculated from dichotomized categories RCB-0/RCB-I versus RCB-II/III, as well as from pCR versus non-pCR. Evaluation was performed using five-fold cross-validation on the training set, the test set is considered a hold-out set.

A descriptive analysis was performed to test the differences in clinicopathological characteristics between the training and test sets. Categorical variables were compared using the ${{{\rm{\chi }}}}^{2}$-test, normal distributed continuous variables were compared using Student’s *t*-tests and other continuous variables using the Mann–Whitney U-test. On the test set, the correlation between the principal components of the deep features and the clinical parameters was assessed using Spearman’s $\rho$. The Kruskal–Wallis test was used to test the association between categorical variables (BI-RADS descriptors and subtype) and the principal components of the deep features, to select features for further exploration. Confidence intervals and comparisons of the AUCs were calculated using DeLong’s method. *p*-values < 0.05 were considered statistically significant.

Statistical tests were performed using Python 3.8.15 with SciPy 1.9.3 and scikit-learn 1.2.2, and ROC analysis using R 4.3.0 with package pROC 1.18.2 to test for statistically significant differences in AUC values on the test set. The package cvAUC 1.1.4 was used to calculate confidence intervals for cross-validated AUC curves on the training set [27, 28].

## Results

### Subjects

**Fig. 2:**
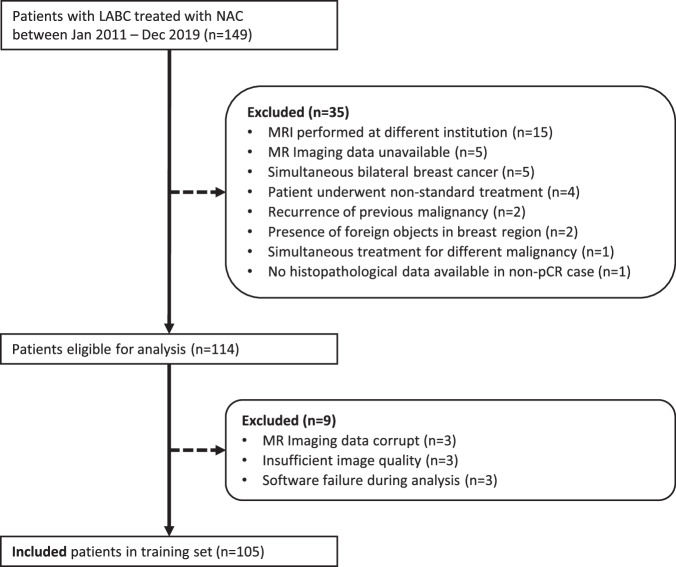
Diagram of patient selection in the training set

**Fig. 3:**
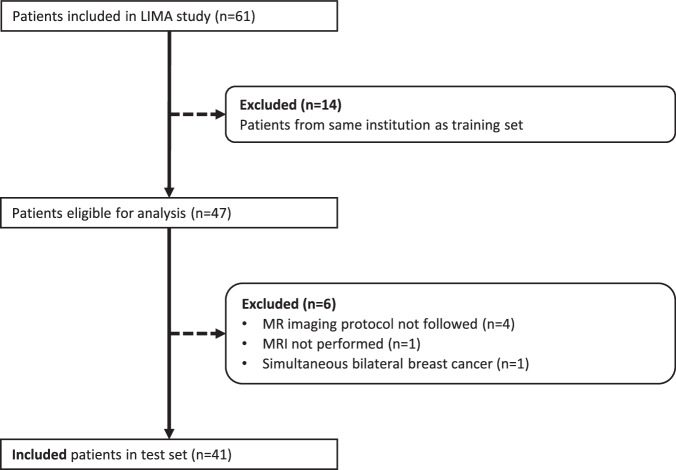
Diagram of patient selection in the test set

One hundred and five patients were included in the training set (Fig. 2), and 41 patients were included in the test set (Fig. 3). The training and test sets showed significant differences in histology, N-stage, tumor grade, subtype, and chemotherapy scheme (Table 2).

**Table 2:** Patient, tumor and treatment characteristics in the training and test sets

Characteristic		Training set (*n* = 105)		Test set (*n* = 41)		*p*-value
		No.	%	No.	%	
Age (years)						
	Mean (min-max)	49.9 (25–73)		52.2 (31–72)		
Laterality						0.56
	Left	49	46.7%	22	53.7%	
	Right	56	53.3%	19	46.3%	
Histology						**0.01**
	Invasive ductal carcinoma/NST	64	61.0%	36	87.8%	
	Invasive ductolobular carcinoma	21	20.0%	1	2.4%	
	Invasive lobular carcinoma	13	12.4%	3	7.3%	
	Other	7	6.7%	1	2.4%	
T-stage						0.59
	T1	15	14.3%	6	14.6%	
	T2	59	56.2%	27	65.9%	
	T3	20	19.0%	7	17.1%	
	T4	9	8.6%	0	0.0%	
	Missing	2	1.9%	1	2.4%	
N-stage						**< 0.01**
	N0	50	47.6%	9	22.0%	
	N1	22	21.0%	23	56.1%	
	N2	14	13.3%	5	12.2%	
	N3	11	10.5%	3	7.3%	
	Missing	8	7.4%	1	2.4%	
M-stage						0.44
	0	92	84.6%	41	100.0%	
	1	4	3.8%	0	0.0%	
	Missing	9	8.6%	0	0.0%	
Grade						**< 0.01**
	Grade 1	12	11.4%	0	0.0%	
	Grade 2	53	50.5%	20	48.8%	
	Grade 3	12	11.4%	21	51.2%	
	Missing	2	1.9%	0	0.0%	
Subtype						**< 0.01**
	ER-/PR-/HER2-	16	15.2%	14	34.1%	
	ER+/HER2-	66	62.9%	15	36.6%	
	HER2+	23	21.9%	12	29.3%	
Surgery type						0.30
	Mastectomy	42	40.0%	21	51.2%	
	Breast-conserving therapy	63	60.0%	20	48.8%	
Chemotherapy scheme						**< 0.01**
	AC + Paclitaxel	33	31.4%	16	39.0%	
	Paclitaxel + carboplatin	2	1.9%	12	29.3%	
	AC + paclitaxel with carboplatin	0	0.0%	13	31.7%	
	FEC + DOC	66	62.9%	0	0.0%	
	Other	4	3.8%	0	0.0%	
RCB score						0.61
	Median (q1–q3)	1.53 (0.77–2.34)		1.59 (0.00–2.51)		
RCB class						
	RCB-0 (pCR)	24	22.9%	13	31.7%	
	RCB-I (minimal RD)	20	19.0%	7	17.1%	
	RCB-II (moderate RD)	52	49.5%	20	48.8%	
	RCB-III (extensive RD)	9	8.6%	1	2.41%	

Missing values were not considered for statistical tests. Significant *p*-values are denoted in bold

*NST* no special type, *ER* estrogen receptor, *PR* progesterone receptor, *HER2* human epidermal growth factor receptor 2, *AC* anthracyclines, *FEC* combination of 5-fluorouracil, epirubicin and cyclophosphamide, *DOC* docetaxel, *RCB* residual cancer burden, *pCR* pathological complete response, *RD* residual disease

### Histopathologic resection specimen evaluation

In the training set, histopathology slides of three patients were not available for revision and calculation of RCB. However, two out of these three patients were reported to have pCR in clinical patient records and were thus assigned RCB score 0. The remaining patient was excluded. In the test set, all histopathological resection specimens were revised. The pCR rate was 22.9% (24/105) in the training set and 31.7% (13/41) in the test set (Table 2). The median RCB scores were not found to be significantly different between the training set (1.53) and test set (1.59) (*p* = 0.61, Mann–Whitney U-test).

### Segmentation performance

The median and interquartile range (IQR, defined as the range spanning the first to third quartile) of the Dice scores of the segmentations in the test set were 0.75 (IQR: 0.27–0.90) for nnU-Net, 0.79 (IQR: 0.33–0.91) for Attention U-Net, and 0.75 (IQR: 0.44–0.87) for the VQED. We show example segmentations and associated ground truths in Supplementary Fig. 1.

### RCB prediction modeling

**Fig. 4:**
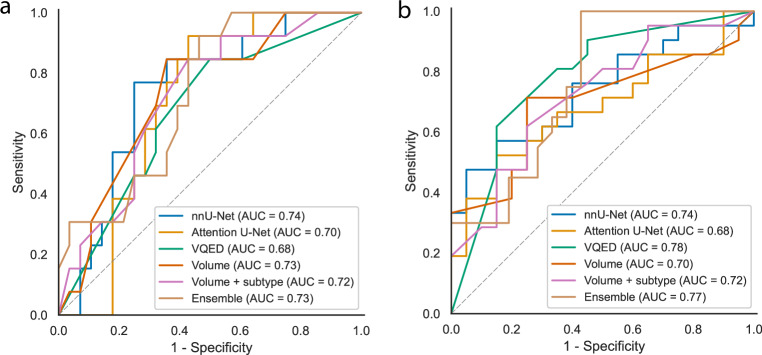
Receiver operator characteristics for predicting pCR (left/**a**) and RCB-0/I (right/**b**) using deep radiomics models based on three different neural networks, the models based on clinical variables only, and an ensemble (combined) model combining the three deep radiomics models with the clinical-radiological volume + subtype model

Cross-validation resulted in the majority of models having optimal performance on the training set with 50 trees and trees with a low maximum depth (Supplementary Table 1). Analysis of the cross-validated performance in the training set showed highly overlapping confidence intervals of AUC for pCR as well as for RCB (Supplementary Table 2). A similar effect was observed in the test set: while the deep radiomics methods had a higher point estimate of AUC, especially for predicting RCB, a statistically significant improvement over clinical-radiological parameters could not be demonstrated in either set. A drop in performance of deep radiomics models compared to clinical-radiological models owing to bad generalization could not be observed either: The clinical-radiological models resulted in AUCs of 0.72–0.74 for predicting pCR, compared to AUCs of 0.68–0.74 of the deep radiomics models, and 0.73 for the ensemble model (Table 3, Fig. 4). In predicting RCB, AUCs varied from 0.70–0.72 for the clinical-radiological models to 0.68–0.79 for the deep radiomics models, the ensemble model had an AUC of 0.77. The Spearman correlation coefficient for the ensemble model was the highest out of the tested models at $R=0.526$.

**Table 3:** Test set performance of deep radiomics models compared to volume-only model

Neural network architecture	AUC pCR (95% CI)	*p*-value	AUC RCB-0/I (95% CI)	*p*-value	Spearman RCB
Clinical-radiological: Volume	0.74 (0.58–0.89)	ref	0.70 (0.53–0.86)	ref	0.467
Clinical-radiological: Volume + subtype	0.72 (0.56–0.88)	0.66	0.72 (0.56–0.88)	0.57	0.475
nnU-Net	0.74 (0.58–0.90)	0.94	0.74 (0.58–0.89)	0.43	0.507
Attention U-Net	0.70 (0.54–0.86)	0.63	0.68 (0.52–0.85)	0.83	0.451
VQED	0.68 (0.51–0.85)	0.66	0.79 (0.64–0.93)	0.42	0.478
Ensemble model	0.73 (0.57–0.88)	0.94	0.77 (0.62–0.91)	0.54	0.526

Statistical tests are performed compared to the volume model

*AUC* area under receiver operator curve, *CI* confidence interval, *pCR* pathological complete response, *RCB* residual cancer burden, *VQED* vector-quantized encoder-decoder

Correlating the principal components of the deep features with clinical parameters showed that several components had moderate or strong correlation ($\left|\rho \right|\ge 0.40$) to pre-NAC tumor volume, post-NAC tumor volume, tumor bed area of residual disease at pathology, and cellularity at pathology (Supplementary Fig. 2).

Analysis of the ensemble model’s weights revealed that most of the contribution to the predictive performance originated from the clinical-radiological model and VQED. The nnU-Net and attention U-Net had no impact on the ensemble model result (Table 4).

**Table 4:** Weights for the combination model in which the predictions of the individual models are taken

Model	Weight
nnU-Net	0.0
Attention U-Net	0.0
VQED	0.198
Volume + subtype	0.554

*VQED* vector-quantized encoder-decoder

There were major differences in the number of utilized feature activations between the CNNs. The most parsimonious model was the VQED, utilizing only 57 deep features condensed into 21 principal components for tumor response modeling. In contrast, the Attention U-Net required over ten thousand features resulting in 78 principal components (Table 5).

**Table 5:** Number of features used for creating the response prediction model

Neural network	Number of deep radiomics features	Deep features with variance > 0.0005 (%)	Principal components (pre, post, delta)
nnU-Net	4800	4783 (99.6%)	35 (13, 8, 14)
Attention U-Net	17,280	15,655 (90.5%)	78 (27, 20, 31)
VQED	1536	57 (3.7%)	21 (8, 4, 9)

The left column shows the number of deep radiomics calculated, the middle column the number of deep radiomics remaining after filtering out radiomics with a < 0.0005 variance, the right-most column shows the number of principal components used for training the RCB prediction model

*RCB* residual cancer burden, *VQED* vector-quantized encoder-decoder

The Kruskal–Wallis test demonstrated that all categorical variables (BI-RADS descriptors and subtype) were associated with at least one deep feature. For each neural network, we selected the five strongest relations between categorical variables. If a variable was associated with multiple deep features, only the strongest association is pursued. This exploratory analysis revealed the distributions of deep features to vary between tumor subtypes, tumor shape/enhancement, and the presence of edema (Supplementary Fig. 3).

## Discussion

We investigated the additional value of deep radiomics compared to the standard clinical-radiological parameters ‘tumor volume’ and ‘subtype,’ for the purpose of assessing the response of locally advanced breast cancer to NAC. We compared the performance of deep features extracted from three different segmentation CNNs and tested if they were complementary to each other and to the clinical-radiological parameters. We observed that the performance of all three networks and the clinical-radiological references was consistently similar: the deep radiomics models had a resulting AUC between 0.68 and 0.74 for predicting pCR and 0.68–0.79 for predicting RCB, while a model using solely tumor volume had an AUC of 0.74 and 0.70 for pCR and RCB, respectively. Combining the deep radiomics and clinical-radiological models did not result in any improvement. The similar performance and absence of complementary benefits suggest that the networks harbor similar information in different representations. Of interest is that several deep features were found to be related to known clinical parameters and BI-RADS descriptors, suggesting they capture information used in current clinical practice.

We compared three segmentation-based neural network architectures, including the widely used nnU-Net, attention U-Net as well as VQED, an auto-encoder method. Leveraging segmentation-based networks enables the re-use of previously trained networks for the purpose of feature extraction without the need for additional labeled data. By extracting feature activation vectors from the coarse resolution bottleneck layer, both tumor and peritumor regions were considered. To evaluate the generalization performance and robustness, we tested our methods on an independent, chronologically separate test set. The test set consisted fully of consecutively included patients from different centers using different MRI equipment. NAC of breast cancer has also seen major developments in the past decade [29]. For example, in the testing cohort, 61% of patients received carboplatin-based regimens, while this was only 2% in the training set. Hence, we estimated the generalization performance over time, considering changes in patient populations due to changes in guidelines and treatments.

Various studies have been performed with the goal of predicting or classifying tumor response to NAC based on breast MRI using radiomics and/or deep learning. An open question in many of these is how the performance generalizes outside the training sets. Ha et al used deep learning to predict both complete and partial responders prior to the initiation of NAC, reporting an AUC of 0.98 for a three-class prediction using the VGG-16 network [30]. Training and validation were performed on single-institution data. Choudhery et al assessed the relation between conventional radiomics and NAC response, investigating both pCR and RCB [31]. They found several features correlated with the RCB index and developed a multivariable model to predict pCR in triple-negative breast cancer patients (AUC = 0.81). Their method required manual tumor segmentations of the axial slice with the largest maximum tumor diameter. While they performed analysis per subtype, they only used single-institution data and acknowledged the need to validate this on external data. Liu et al used SAGS-net to predict pCR in two publicly available datasets, achieving cross-validated AUCs of 0.93 on both datasets separately. The generalization performance of the models, by training on one dataset and testing on the other, was not performed [32]. Granzier et al used conventional radiomics to predict pCR but reported poor performance (AUC = 0.50–0.55) when applying the model on data from a different institution, indicating poor generalization [11]. Finally, Li et al used three tumor-related features as well as breast parenchymal enhancement to predict pCR in the large multicenter I-SPY2 cohort, reporting an AUC of 0.81 (95% CI: 0.76–0.86) to predict pCR [33]. Hold-out validation on a per study-site base was not performed and the method is not applicable to cases exhibiting a radiological complete response. In our study, we evaluated generalizability extensively using a multi-institution, multi-vendor, and chronologically separate dataset and considered both pCR and RCB categories as endpoints for response. We did not observe any difference in generalization between clinical-radiological and deep radiomics models.

This study had several limitations. First, the limited sample size of the test set precluded meaningful analysis of differences in performance between the different neural networks or different subtypes. Validation in a larger cohort could provide more statistical power and allow meaningful analysis on a subtype level. Although we followed best practices to minimize the risk of overfitting, such as using data augmentation for the segmentation CNNs and the use of dimensionality reduction, the risk of overfitting cannot be completely mitigated. It was not considered feasible to augment MRI and RCB simultaneously in a consistent manner because the interaction between the two is currently unknown. Although we were unable to demonstrate significant differences in the performance of deep features compared to standard clinical predictors—observing only small differences in AUC with highly overlapping confidence intervals—we cannot exclude that more subtle differences will become apparent at a larger sample size. Second, the cohorts had slightly different inclusion criteria: The test set excluded luminal A breast cancers, some of which were included in the training cohort. Luminal A types are known to respond poorly to chemotherapy, and this might have influenced response prediction [34]. Oligometastatic disease treated with curative intent was also not included in the test set. Third, we only considered segmentation-derived deep features from the bottleneck layer in our research. Neural networks directly trained for the task of RCB prediction may contain more informative features and potentially improve RCB prediction performance. This requires a different training approach and does not allow the re-use of pre-trained networks. An advantage of our method is that it does allow the re-use of pre-trained networks and can be retargeted to different biomarkers or predictors without the time-consuming retraining of a CNN.

The innovative aspects and potential advantages of this work are the use of image segmentation networks to leverage highly dimensional neural networks when study sample sizes are relatively small. In the current study, for instance, data on MRI and corresponding RCB scores are relatively scarce. The reported approach allows the use of retrospective datasets for which segmentations are already available. A similar concept is used by foundation models, which are trained on extremely large datasets [35]. The embeddings generated by those models are then used for downstream tasks such as segmentation, classification, or text generation. Our approach is comparable, except that the embeddings are extracted from the bottleneck layer of a segmentation network and therefore require fundamentally less training data than foundation models. Hence, it is suitable for smaller datasets, requires less computation, and can leverage a vast library of pre-trained segmentation networks that are already available. Another advantage of our method is that it could provide complementary information if reliable tumor size measurements cannot be performed (e.g., in cases with high background parenchymal enhancement) or if subtype cannot reliably be determined (e.g., in cases where biopsies do not accurately sample all subtypes present). It is also observer-independent, thus reducing potential inter-observer variation between radiologists.

Based on our findings, we propose the following directions for future research. First, training and validation on larger cohorts, although concurrent availability of RCB and MRI is currently sparse. This will allow a more detailed analysis of performance, e.g., per subtype. Second, using methods to directly predict RCB based on imaging, without a segmentation step in between, is of considerable interest. This will also require larger datasets, as data augmentation of imaging and RCB data combined is considered challenging. Third, we only investigated the use of deep features extracted from the bottleneck layer. While we consider the bottleneck layer to be the most parsimonious layer, combining it with deep features extracted from intermediate layers could potentially lead to more detailed representations of tumor characteristics. Finally, we only investigated the use of deep radiomics for the prediction of response to NAC in breast cancer patients. Using the methodology for different clinical problems can lead to more insights into the general performance of deep features across domains.

## Conclusion

This study shows that deep radiomics extracted from commonly used segmentation networks contained similar information to tumor volume and subtype for the purposes of inferring pCR and RCB after NAC. We could not show that deep radiomics complements or improves upon these standard clinical features. Deep radiomic features had similar generalizing performance in an independent test set compared to standard clinical parameters but consistently did not outperform the clinical parameters, nor provide substantial complementary information in the LIMA trial.

## Supplementary information

ELECTRONIC SUPPLEMENTARY MATERIAL

## Data Availability

Source code related to the deep radiomics extraction is available at https://github.com/Lab-Translational-Cancer-Imaging/Deep_radiomics_extraction. The datasets analyzed during the current study are not publicly available due to patient privacy but are available at the corresponding author on reasonable request.

## Abbreviations

AC: Anthracyclines
AUC: Area under the receiver operating characteristics curve
BI-RADS: Breast Imaging-Reporting and Data System
CI: Confidence interval
CNN: Convolutional neural network
DCE-MRI: Dynamic contrast-enhanced magnetic resonance imaging
DOC: Docetaxel
ER: Estrogen receptor
HER2: Human epidermal growth factor receptor 2
IQR: Interquartile range
IRB: Institutional review board
LIMA: Liquid biopsies and IMAging study
NAC: Neoadjuvant chemotherapy
PCA: Principal component analysis
pCR: Pathological complete response
RCB: Residual cancer burden
RD: Residual disease
TIL: Tumor-infiltrating lymphocyte
TN: Triple negative
UMC Utrecht: University Medical Center Utrecht
VQED: Vector-quantized encoder-decoder
