# Supplementary material for: Deep learning-based radiomics does not improve residual cancer burden prediction post-chemotherapy in LIMA breast MRI trial
Source: Eur Radiol. 2025 Aug 6;36(2):850–62. doi: 10.1007/s00330-025-11801-z (PMC12953280; doi:10.1007/s00330-025-11801-z)
Supplement: Supplementary file 1 — ELECTRONIC SUPPLEMENTARY MATERIAL [file 330_2025_11801_MOESM1_ESM.pdf]

Deep learning-based radiomics does not improve residual cancer prediction burden post-chemotherapy in LIMA breast MRI trial

ELECTRONIC SUPPLEMENTARY MATERIAL

Neural network training details

Attention U-Net

Data augmentations used during training were random scaling of the image (scaling factor sampled from a normal distribution with $\mu = 1; \sigma = 0.1$, random rotations (uniform randomly sampled between -5° and +5°) and horizontal flipping (chance of 50%). The batch size used was four. The initial depth of the network was set to be 72 layers. Cross-entropy was used for the loss function. The Adam optimizer was used with an initial learning rate of 1e-5 [1]. The learning rate was decreased by a factor 10 after 12 epochs if the loss not decreasing by at least 10e-7 and early-stopping was set to 19 epochs of no loss decrease by more than 10e-7.

Deep feature extraction details

nnU-Net

A modified version of nnU-Net was used to extract the deep features from the trained network at inference time, as illustrated in [2]. Voxels corresponding to the segmented tumor were selected in the bottleneck layer and the values of the deep features corresponding to these voxels were extracted. For each deep feature, its value was sampled at five different percentiles (10th, 25th, 50th, 75th and 90th). The feature activation vector for each scan was then composed by concatenating these percentiles for every deep feature into one vector, resulting in a feature activation vector of 1600 elements per examination (as the network contains 320 deep features). If there was an empty tumor segmentation (i.e. due to radiological complete response), deep features were assigned the value zero.

Attention U-Net

The Attention U-Net feature activation vector was extracted using a similar procedure to that for nnU-Net, in which deep features were extracted after inference based on the tumor segmentation. The network contains 1152 deep features. The values of each deep feature were sampled at five percentiles (10th, 25th, 50th, 75th, 90th), resulting in a feature activation vector of 5760 elements per examination. Similar to the nnU-Net, cases with an empty segmentation were assigned zero values for their deep features.

Vector-quantized encoder-decoder

The VQED network expresses deep features in the form of a 512-bin histogram per slice, where each bin corresponds to one feature. The feature activation vector is calculated by summing these histograms elementwise for all slices in an examination. Unlike the U-Net-derived networks, VQED calculates deep features for the entire scan, not just the tumor area and is therefore not affected by tumor segmentations.

RCB prediction model details

Three deep radiomics RCB prediction models were trained, one for each neural network and associated set of deep features. The three radiomics followed the same training procedure. First, features with a variance less than 0.0005 (i.e. “near-invariant features”) were first removed from the feature activation vectors, followed by principal component analysis (PCA)

applied separately to the pre-NAC, post-NAC, and delta-NAC feature activation vectors. The minimum cumulative variance was chosen to retain at least five components per examination, with the selected threshold being the lowest of either 80%, 90%, 95% or 99%. The Yeo-Johnson transformation was applied to RCB scores before fitting the model to account for the non-normal distribution [3]. Optimal hyperparameters for the random forest regression model were then determined using five-fold cross-validation on the training set: optimal hyperparameters were chosen to maximize AUC$(\mu - \sigma)$, μ being the mean AUC over all folds and σ the standard deviation of the AUCs as to ensure minimum variation in performance per fold. Note that features were not harmonized with regards to scanner manufacturer, cohort or any other factor.

Supplementary tables

Supplementary Table 1: Selected hyperparameters after five-fold cross-validation on the training set. Numbers below 1 indicate fractions of data, while integers denote absolute numbers of samples. No. trees: number of trees in the random forest. Max tree depth: maximum depth of the tree, Min samples split: minimum number of samples that are required before a node is split. Min samples leaf: Minimum number of samples required before a node can be considered a leaf node. Labels and descriptions of these parameters courtesy of scikit-learn [4].

**Model**	**No trees**	**Max tree depth**	**Max samples split**	**Min samples leaf**
Volume	50	4	0.3	1
Volume + subtype	50	2	16	0.1
nnU-Net	50	2	16	0.1
Attention U-Net	20	No max	4	1
VQED	50	1	2	0.2

Supplementary Table 2: Training set performance of deep radiomics models compared to clinical-radiological models after five-fold cross-validation. Numbers between brackets are 95% confidence intervals. pCR: Pathological complete response. VQED: Vector-quantized encoder decoder. Responders are defined as patients exhibiting pCR or RCB-I.

**Neural network or model**	**AUC pCR**	**AUC Responders**
Volume	0.845 (0.760 – 0.933)	0.775 (0.673 – 0.877)
Volume + subtype	0.892 (0.821 – 0.964)	0.826 (0.734 – 0.918)
Ensemble	0.893 (0.825 – 0.961)	0.815 (0.728 – 0.902)
nnU-Net	0.795 (0.702 – 0.889)	0.733 (0.634 – 0.832)
Attention U-Net	0.820 (0.711 – 0.929)	0.739 (0.639 – 0.840)
VQED	0.786 (0.678 – 0.892)	0.707 (0.606 – 0.807)

Supplementary Figures

Figure S1: Illustration of three example cases and associated model predictions. Each column represents one patient. From top to bottom: Post-contrast subtraction of patient before neoadjuvant chemotherapy (NAC) with ground truth tumor segmentation in purple outline, post-contrast subtraction after NAC at a similar position, output of nnU-Net segmentation, output of Attention U-Net segmentation, output of VQED network, and description of the case.

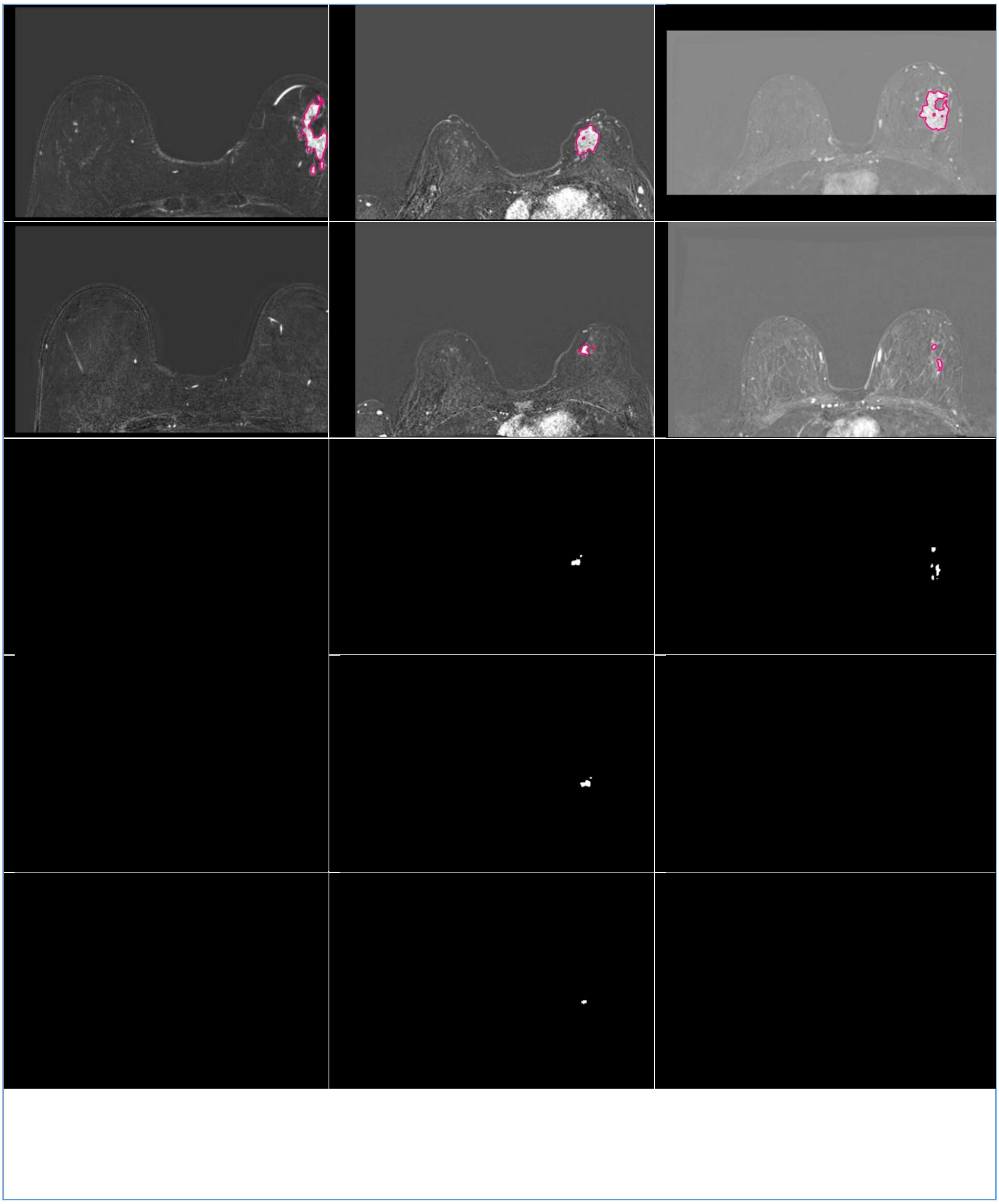

41 year-old patient with TN breast cancer. Radiological assessment resulted in a rCR. Post-surgery, the patient had a pCR and associated RCB score of 0.<br>All networks assessed the case as a complete responder. The combined deep radiomics model predicted a good response (RCB-0/I).<br>The patient underwent a mastectomy.	57yo with ER+/PR+/HER2- breast cancer. Radiological assessment resulted in residual tumor. Post-surgery, the patient had an RCB score of 2.1 (category RCB-II, bad responder).<br>All networks detected residual tumor. The deep radiomics model assessed the case as a bad response (RCB-II/III).<br>The patient underwent a mastectomy.	45yo, ER-/PR-/HER2+. Radiological assessment showed residual tumor. Post-surgery, the patient had a pCR and associated RCB score of 0.<br>Only the nnU-Net recorded residual tumor, the other two networks did not detect residual cancer.<br>The patient underwent a lumpectomy.

Abbreviations:

TN: Triple Negative
ER: Estrogen Receptor
PR: Progesteron Receptor
HER2: Human Epidermal growth factor Receptor 2
RCB: Residual Cancer Burden
rCR: radiological Complete Response
pCR: pathological Complete Response
VQED: Vector-quantized encoder-decoder

Supplementary Figure 2: Correlation of principal components of deep features with clinical parameters

Caption: Heatmap showing Spearman’s correlation between continuous biological/clinical parameters and principal components from the deep features. Numbers in each cell correspond to Spearman’s correlation between the principal component and biological/clinical parameter. Moderate correlations ($|\rho| \geq 0.40$) shown in white, strong correlations ($|\rho| \geq 0.60$) displayed in **boldface**.

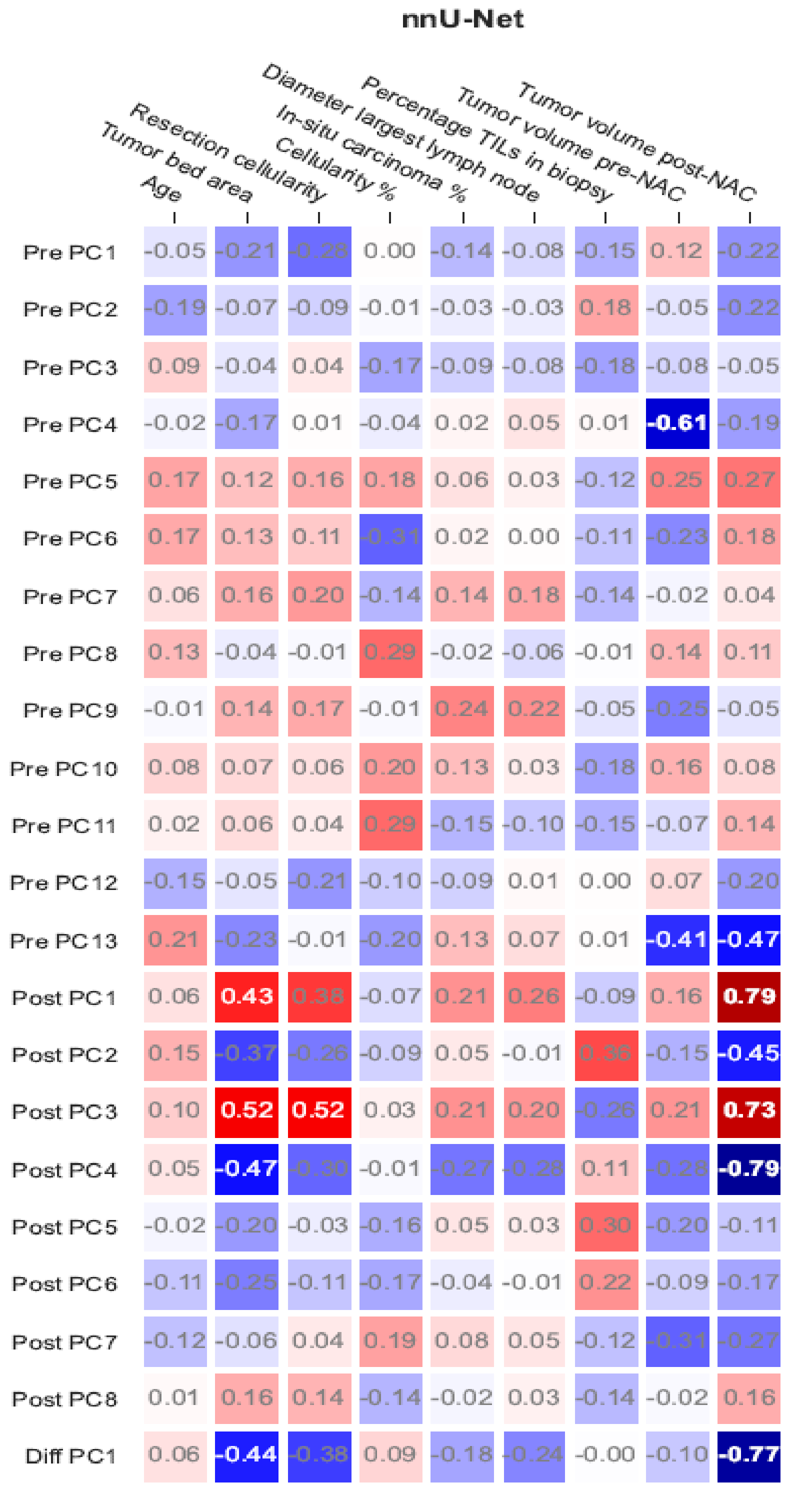

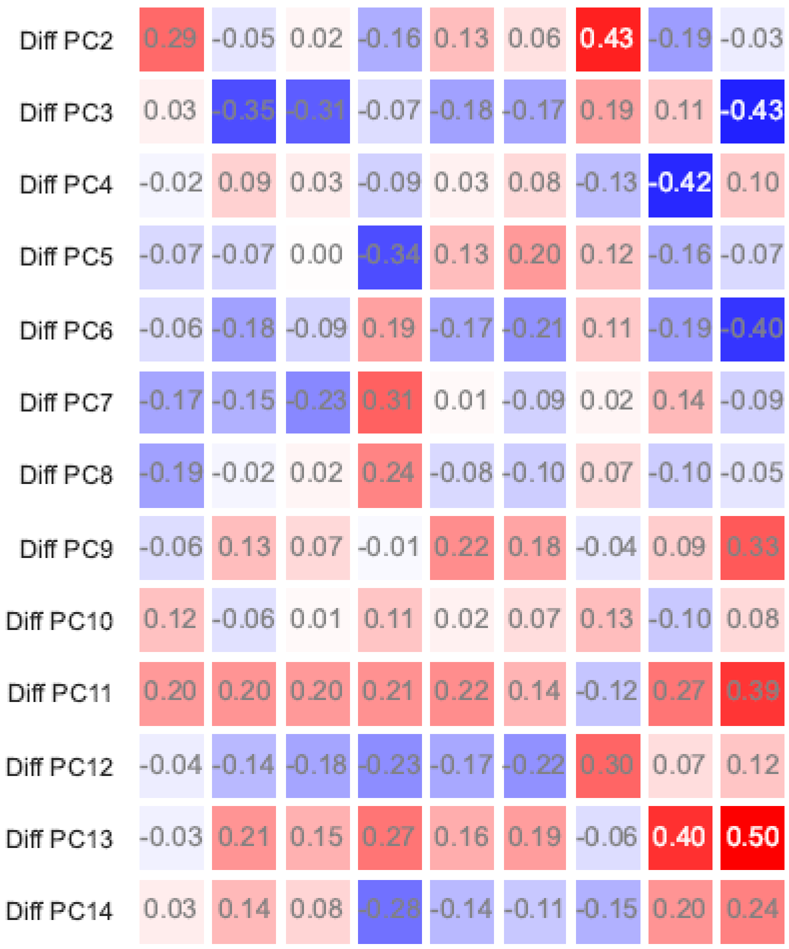
Diff PC2 0.29 -0.05 0.02 -0.16 0.13 0.06 0.43 -0.19 -0.03
Diff PC3 0.03 -0.35 -0.31 -0.07 -0.18 -0.17 0.19 0.11 -0.43
Diff PC4 -0.02 0.09 0.03 -0.09 0.03 0.08 -0.13 -0.42 0.10
Diff PC5 -0.07 -0.07 0.00 -0.34 0.13 0.20 0.12 -0.16 -0.07
Diff PC6 -0.06 -0.18 -0.09 0.19 -0.17 -0.21 0.11 -0.19 -0.40
Diff PC7 -0.17 -0.15 -0.23 0.31 0.01 -0.09 0.02 0.14 -0.09
Diff PC8 -0.19 -0.02 0.02 0.24 -0.08 -0.10 0.07 -0.10 -0.05
Diff PC9 -0.06 0.13 0.07 -0.01 0.22 0.18 -0.04 0.09 0.33
Diff PC10 0.12 -0.06 0.01 0.11 0.02 0.07 0.13 -0.10 0.08
Diff PC11 0.20 0.20 0.20 0.21 0.22 0.14 -0.12 0.27 0.39
Diff PC12 -0.04 -0.14 -0.18 -0.23 -0.17 -0.22 0.30 0.07 0.12
Diff PC13 -0.03 0.21 0.15 0.27 0.16 0.19 -0.06 0.40 0.50
Diff PC14 0.03 0.14 0.08 -0.28 -0.14 -0.11 -0.15 0.20 0.24

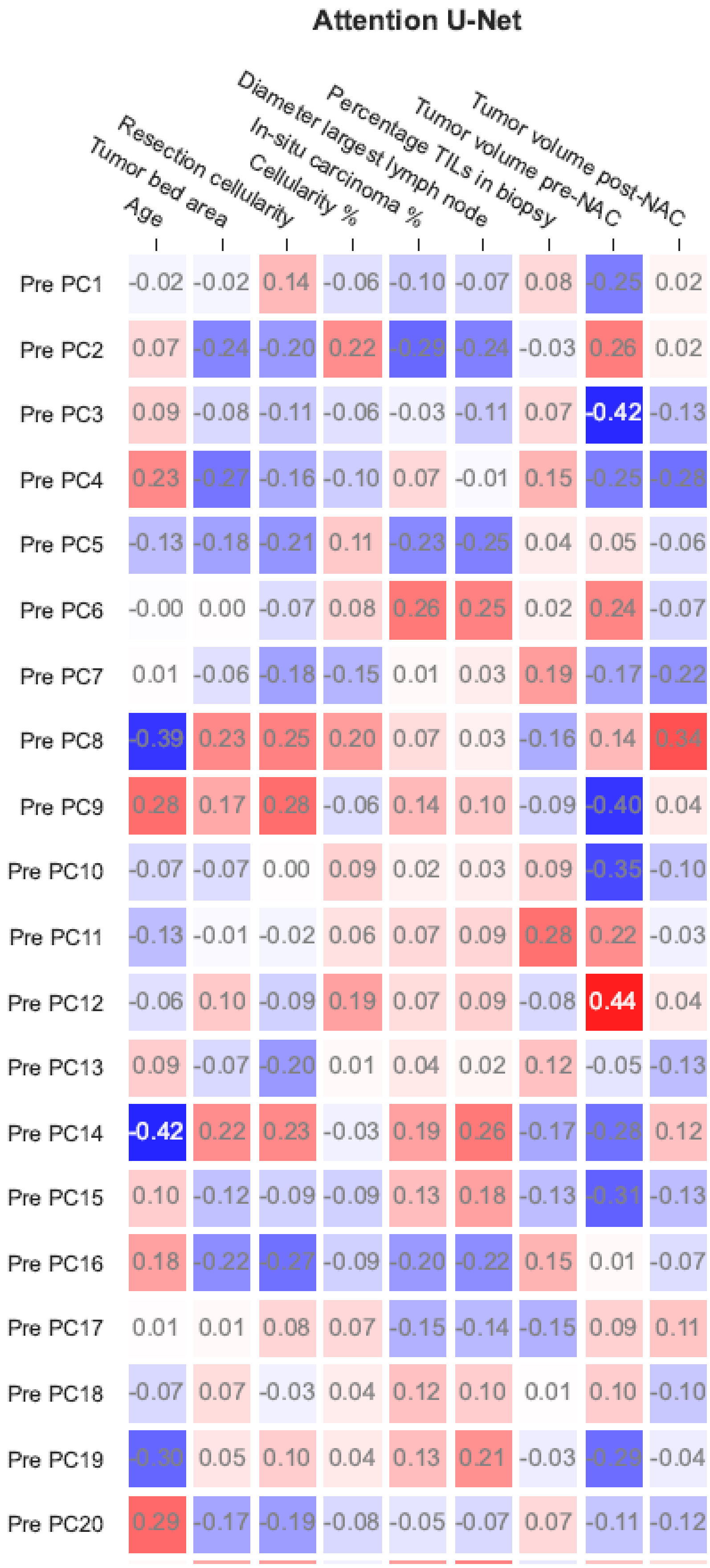
Attention U-Net
Age
Tumor bed area
Resection cellularity
Cellularity %
In-situ carcinoma %
Diameter largest lymph node
Percentage TILs in biopsy
Tumor volume pre-NAC
Tumor volume post-NAC
Pre PC1 -0.02 -0.02 0.14 -0.06 -0.10 -0.07 0.08 -0.25 0.02
Pre PC2 0.07 -0.24 -0.20 0.22 -0.29 -0.24 -0.03 0.26 0.02
Pre PC3 0.09 -0.08 -0.11 -0.06 -0.03 -0.11 0.07 -0.42 -0.13
Pre PC4 0.23 -0.27 -0.16 -0.10 0.07 -0.01 0.15 -0.25 -0.28
Pre PC5 -0.13 -0.18 -0.21 0.11 -0.23 -0.25 0.04 0.05 -0.06
Pre PC6 -0.00 0.00 -0.07 0.08 0.26 0.25 0.02 0.24 -0.07
Pre PC7 0.01 -0.06 -0.18 -0.15 0.01 0.03 0.19 -0.17 -0.22
Pre PC8 -0.39 0.23 0.25 0.20 0.07 0.03 -0.16 0.14 0.34
Pre PC9 0.28 0.17 0.28 -0.06 0.14 0.10 -0.09 -0.40 0.04
Pre PC10 -0.07 -0.07 0.00 0.09 0.02 0.03 0.09 -0.35 -0.10
Pre PC11 -0.13 -0.01 -0.02 0.06 0.07 0.09 0.28 0.22 -0.03
Pre PC12 -0.06 0.10 -0.09 0.19 0.07 0.09 -0.08 0.44 0.04
Pre PC13 0.09 -0.07 -0.20 0.01 0.04 0.02 0.12 -0.05 -0.13
Pre PC14 -0.42 0.22 0.23 -0.03 0.19 0.26 -0.17 -0.28 0.12
Pre PC15 0.10 -0.12 -0.09 -0.09 0.13 0.18 -0.13 -0.31 -0.13
Pre PC16 0.18 -0.22 -0.27 -0.09 -0.20 -0.22 0.15 0.01 -0.07
Pre PC17 0.01 0.01 0.08 0.07 -0.15 -0.14 -0.15 0.09 0.11
Pre PC18 -0.07 0.07 -0.03 0.04 0.12 0.10 0.01 0.10 -0.10
Pre PC19 -0.30 0.05 0.10 0.04 0.13 0.21 -0.03 -0.29 -0.04
Pre PC20 0.29 -0.17 -0.19 -0.08 -0.05 -0.07 0.07 -0.11 -0.12

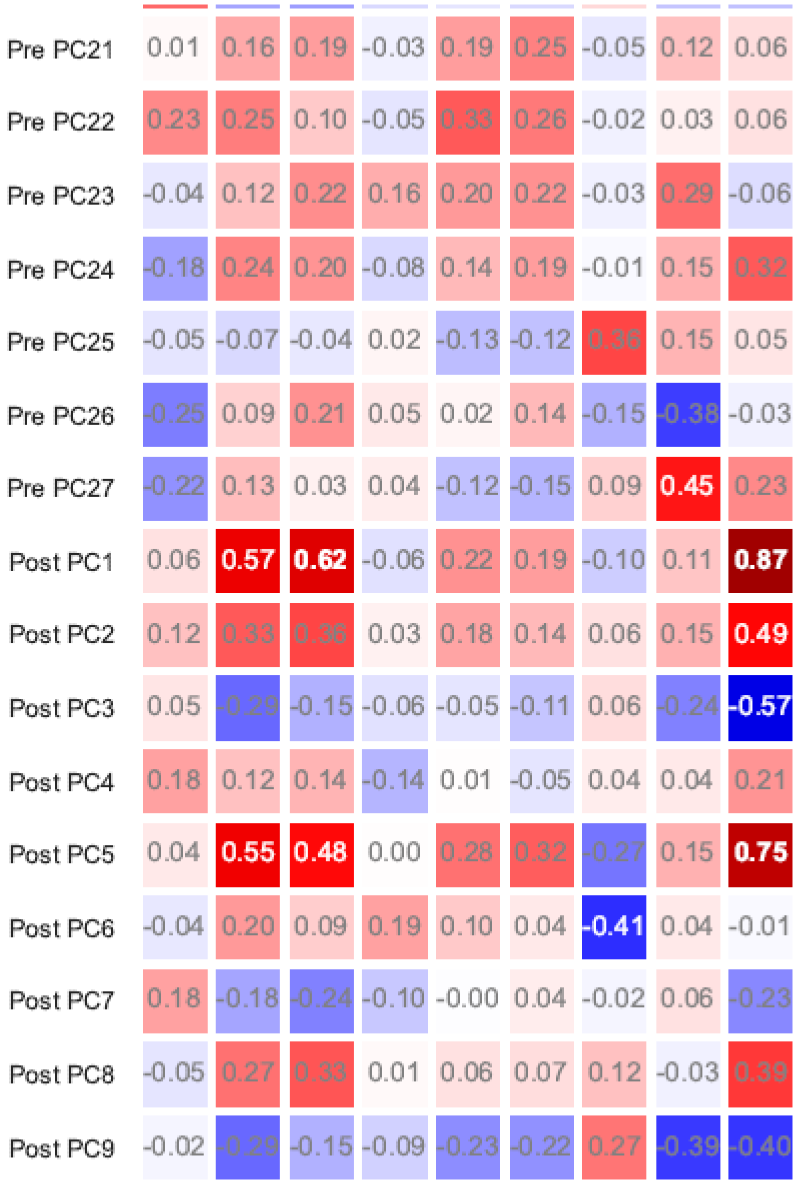

Pre PC21 0.01 0.16 0.19 -0.03 0.19 0.25 -0.05 0.12 0.06
Pre PC22 0.23 0.25 0.10 -0.05 0.33 0.26 -0.02 0.03 0.06
Pre PC23 -0.04 0.12 0.22 0.16 0.20 0.22 -0.03 0.29 -0.06
Pre PC24 -0.18 0.24 0.20 -0.08 0.14 0.19 -0.01 0.15 0.32
Pre PC25 -0.05 -0.07 -0.04 0.02 -0.13 -0.12 0.36 0.15 0.05
Pre PC26 -0.25 0.09 0.21 0.05 0.02 0.14 -0.15 -0.38 -0.03
Pre PC27 -0.22 0.13 0.03 0.04 -0.12 -0.15 0.09 0.45 0.23
Post PC1 0.06 0.57 0.62 -0.06 0.22 0.19 -0.10 0.11 0.87
Post PC2 0.12 0.33 0.36 0.03 0.18 0.14 0.06 0.15 0.49
Post PC3 0.05 -0.29 -0.15 -0.06 -0.05 -0.11 0.06 -0.24 -0.57
Post PC4 0.18 0.12 0.14 -0.14 0.01 -0.05 0.04 0.04 0.21
Post PC5 0.04 0.55 0.48 0.00 0.28 0.32 -0.27 0.15 0.75
Post PC6 -0.04 0.20 0.09 0.19 0.10 0.04 -0.41 0.04 -0.01
Post PC7 0.18 -0.18 -0.24 -0.10 -0.00 0.04 -0.02 0.06 -0.23
Post PC8 -0.05 0.27 0.33 0.01 0.06 0.07 0.12 -0.03 0.39
Post PC9 -0.02 -0.29 -0.15 -0.09 -0.23 -0.22 0.27 -0.39 -0.40

Post PC10	0.11	-0.32	-0.23	-0.03	-0.18	-0.22	0.18	-0.24	-0.60
Post PC11	-0.28	-0.46	-0.39	-0.11	-0.25	-0.18	0.45	-0.27	-0.37
Post PC12	0.19	-0.07	-0.08	-0.10	0.01	0.02	0.22	0.12	-0.03
Post PC13	0.24	-0.19	-0.15	-0.10	0.09	0.09	0.18	-0.11	-0.34
Post PC14	0.06	0.02	0.04	0.13	0.21	0.13	-0.12	0.02	-0.07
Post PC15	-0.09	-0.15	-0.11	-0.11	-0.19	-0.13	0.31	-0.26	-0.23
Post PC16	-0.19	0.25	0.22	0.06	-0.14	-0.07	0.01	0.18	0.45
Post PC17	-0.02	0.16	0.20	0.07	0.20	0.26	-0.30	0.23	0.35
Post PC18	-0.13	-0.03	0.01	-0.20	-0.12	-0.06	0.36	-0.09	0.18
Post PC19	-0.23	-0.08	0.02	0.14	-0.16	-0.19	0.02	-0.28	-0.21
Post PC20	0.00	-0.43	-0.48	0.03	-0.23	-0.19	-0.05	-0.03	-0.60
Diff PC1	0.11	0.49	0.52	-0.18	0.31	0.24	-0.03	0.10	0.64
Diff PC2	-0.09	0.01	-0.00	-0.14	0.20	0.17	-0.03	-0.21	-0.18
Diff PC3	-0.06	0.33	0.44	-0.23	0.21	0.23	0.21	-0.06	0.29
Diff PC4	0.01	0.10	0.03	-0.03	-0.04	-0.03	0.16	0.03	0.40
Diff PC5	-0.19	0.06	0.13	0.11	-0.16	-0.18	-0.04	0.12	0.18
Diff PC6	0.31	0.12	0.05	-0.18	0.13	0.14	0.06	0.01	0.09
Diff PC7	0.02	-0.18	0.10	-0.11	-0.10	-0.05	0.12	0.09	0.01
Diff PC8	-0.14	0.27	0.32	-0.13	0.02	0.05	-0.15	0.12	0.35
Diff PC9	0.04	-0.18	-0.12	-0.07	-0.26	-0.16	0.00	-0.30	-0.28
Diff PC10	-0.09	-0.15	-0.21	-0.07	-0.09	0.02	0.00	0.01	-0.01
Diff PC11	0.03	-0.09	-0.09	-0.09	-0.23	-0.13	-0.11	-0.09	-0.01
Diff PC12	-0.07	0.19	0.19	0.05	0.22	0.13	-0.30	-0.26	0.16
Diff PC13	-0.07	-0.07	-0.03	0.23	0.04	0.09	0.22	-0.09	0.08
Diff PC14	0.13	-0.09	-0.05	-0.19	0.08	0.19	-0.01	-0.23	-0.02

Diff PC15	0.19	-0.41	-0.38	-0.05	-0.16	-0.22	0.37	-0.07	-0.45
Diff PC16	0.39	-0.16	-0.10	0.02	0.07	0.10	-0.29	-0.10	-0.14
Diff PC17	0.25	-0.06	-0.07	-0.33	-0.05	-0.05	0.09	-0.38	-0.32
Diff PC18	-0.11	0.05	0.11	-0.03	-0.03	-0.10	0.16	0.19	0.15
Diff PC19	-0.03	0.01	-0.02	-0.14	0.11	0.10	-0.06	-0.13	0.01
Diff PC20	0.21	-0.05	-0.18	0.03	-0.15	-0.14	0.12	0.15	-0.09
Diff PC21	0.23	0.10	0.09	-0.11	0.29	0.28	0.20	0.34	0.14
Diff PC22	-0.00	0.29	0.45	0.06	0.27	0.20	-0.16	0.00	0.27
Diff PC23	0.17	-0.23	-0.22	-0.08	-0.10	-0.07	0.09	0.01	-0.18
Diff PC24	0.02	-0.29	-0.36	0.04	-0.04	-0.02	0.24	-0.24	-0.42
Diff PC25	-0.32	-0.18	0.04	-0.05	-0.10	-0.08	0.05	-0.54	-0.27
Diff PC26	0.05	-0.04	-0.23	-0.14	-0.21	-0.17	0.18	0.12	-0.03
Diff PC27	0.06	-0.02	0.13	0.04	0.12	0.18	-0.18	-0.26	-0.09
Diff PC28	-0.17	0.24	0.24	0.10	0.22	0.27	-0.02	0.00	0.23
Diff PC29	-0.04	0.01	-0.11	-0.05	-0.18	-0.08	0.19	0.33	0.21
Diff PC30	-0.11	0.13	0.15	-0.11	0.05	0.03	-0.14	0.08	0.17
Diff PC31	-0.10	0.06	0.05	0.05	0.00	-0.09	0.02	0.16	0.16

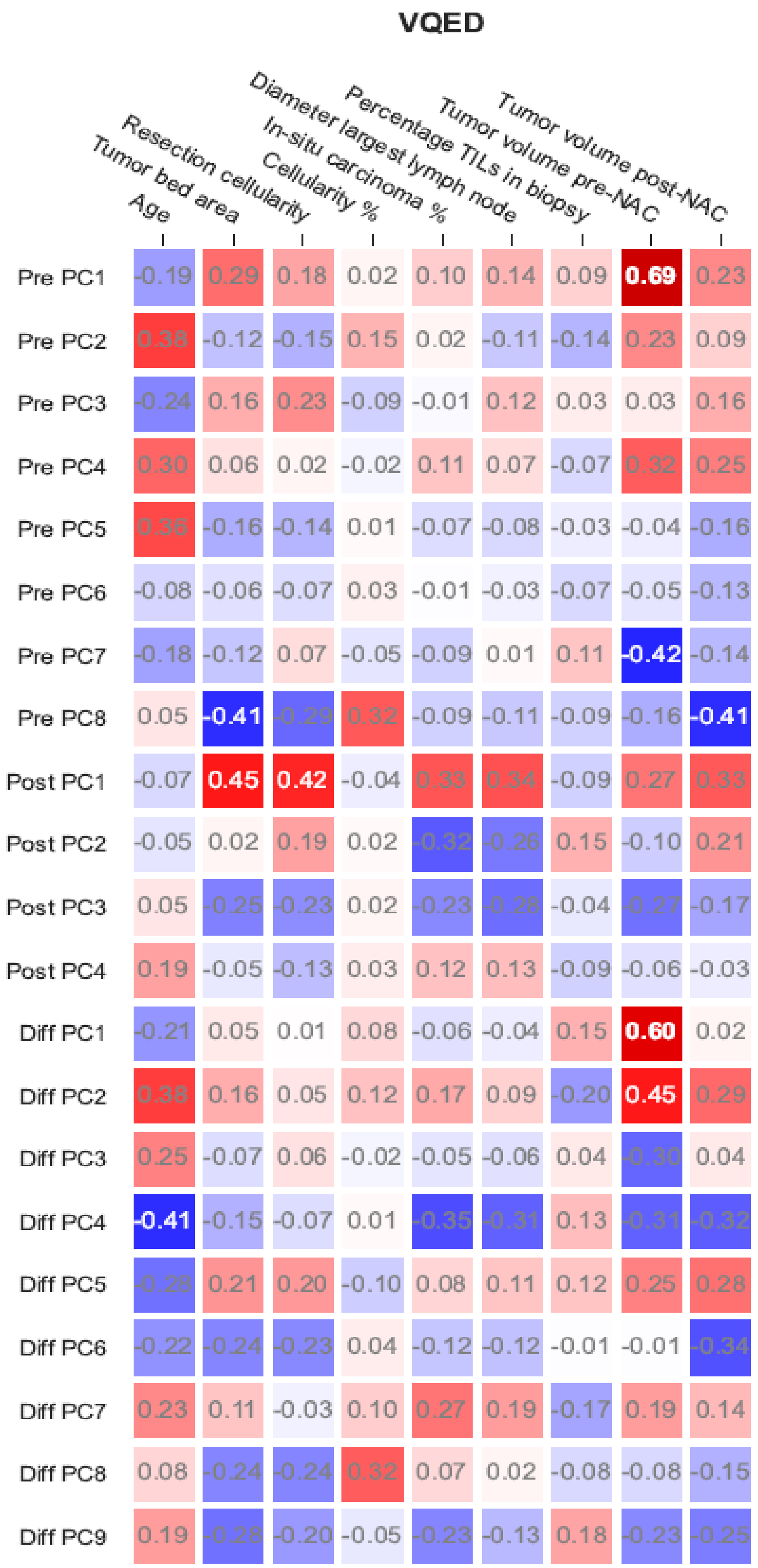

NAC: Neoadjuvant Chemotherapy, PC: Principal Component, TILs: Tumor Infiltrating Lymphocytes.

Supplementary Figure 3: Exploratory analysis of deep features in relation to qualitative features. Each column corresponds to a neural network. The boxplots show one deep feature related to one BI-RADS descriptor or tumor subtype.

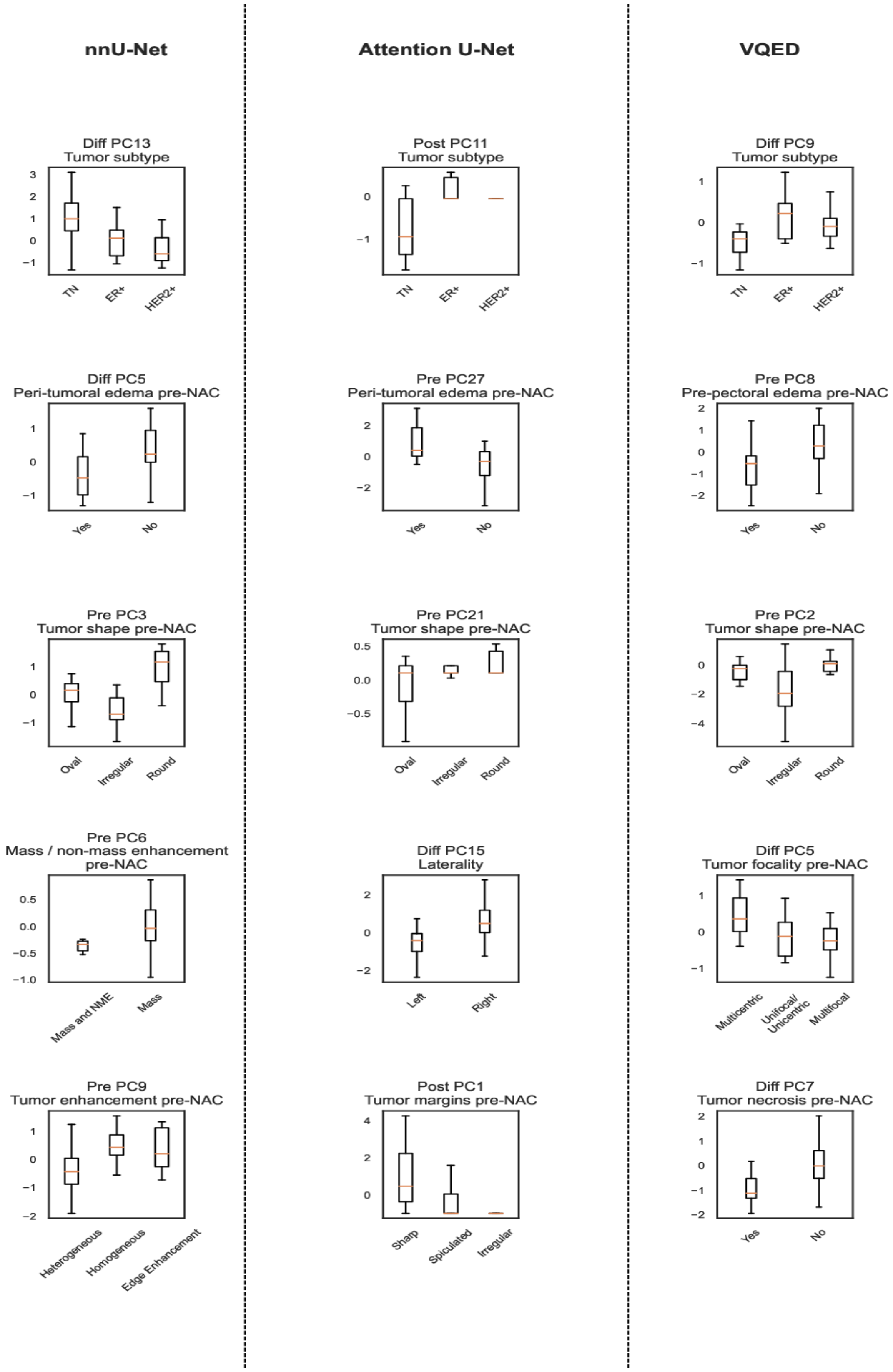

PC: Principal component, NAC: neoadjuvant chemotherapy, NME: Non-mass enhancement.

Supplemental bibliography

1. Kingma DP, Ba LJ (2015) Adam: A Method for Stochastic Optimization

2. Isensee F, Jaeger PF, Kohl SAA, et al (2021) nnU-Net: a self-configuring method for deep learning-based biomedical image segmentation. Nat Methods 18:203–211. https://doi.org/10.1038/s41592-020-01008-z

3. Yeo I-K (2000) A new family of power transformations to improve normality or symmetry. Biometrika 87:954–959. https://doi.org/10.1093/biomet/87.4.954

4. Pedregosa F, Varoquaux G, Gramfort A, et al (2011) Scikit-learn: Machine Learning in Python. J Mach Learn Res 12:2825–2830
